# IDH1/2 mutation status combined with Ki-67 labeling index defines distinct prognostic groups in glioma

**DOI:** 10.18632/oncotarget.4920

**Published:** 2015-08-18

**Authors:** Ailiang Zeng, Qi Hu, Yanwei Liu, Zheng Wang, Xiaoming Cui, Rui Li, Wei Yan, Yongping You

**Affiliations:** ^1^ Department of Neurosurgery, The First Affiliated Hospital of Nanjing Medical University, Nanjing 210029, PR China; ^2^ Beijing Neurosurgical Institute, Capital Medical University, Beijing 100050, PR China

**Keywords:** IDH1/2 mutation, Ki-67 labeling index, prognosis, glioma, molecular classification

## Abstract

The current World Health Organization (WHO) classification of human gliomas is mainly based on morphology. However, it has limitations in prognostic prediction. We examined whether combining isocitrate dehydrogenase (IDH) 1/2 mutation status with the Ki-67 labeling index would improve the definition of prognostically distinct entities. We investigated the correlation of Ki-67 expression with IDH1/2 mutation status and their impact on clinical outcome in 703 gliomas. Low Ki-67 expression closely overlapped with IDH1/2 mutation in our cohort (*P* < 0.0001). Patients with IDH1/2 mutation survived significantly longer than patients with wild-type IDH1/2 did (*P* < 0.0001); higher Ki-67 expression was associated with shorter progression-free survival and overall survival (OS) (*P* < 0.0001). IDH1/2 combined with Ki-67 was used to re-classify glioma patients into five groups. IDH1/2 mutant patients with low and moderate Ki-67 expression (Group1) had the best prognosis, whereas patients with wild-type IDH1/2 and high Ki-67 expression (Group5) had the worst prognosis (Median OS = 1527 vs. 355 days, *P* < 0.0001). To summarize, our new classification model distinguishes biologically distinct subgroups and provides prognostic information regardless of the conventional WHO grade. Classification based on IDH1/2 mutation status and Ki-67 expression level could be more convenient for clinical application and guide personalized treatment in malignant gliomas.

## INTRODUCTION

Diffuse gliomas represent a biologically heterogeneous group of primary brain tumors [1]. Their intrinsically invasive characteristic encumbers complete surgical resection, mandating the development of more effective medical therapies [2]. World Health Organization (WHO) grade I gliomas are often curable with definitive surgical resection [3]; WHO grade II, III, and IV gliomas are malignant, diffuse, and with poor outcome; glioblastoma (WHO grade IV, the most invasive grade) is characterized by astrocytic morphology with induced angiogenesis and/or necrosis. The ability of the current WHO classification for predicting human glioma diagnosis and prognosis remains limited [4, 5]. In the past 30 years, many molecules have been implicated in the grading of prognostically different astrocytic tumors [6]. In 2009, it was determined that isocitrate dehydrogenase (IDH) 1/2 mutations were correlated with a prognostically improved adult glioma subtype. IDH1 mutation affects amino acid 132 of the *IDH1* gene. The analogous amino acid (R172) of the *IDH2* gene is often affected in tumors without IDH1 mutations. IDH1 mutation is involved in >70% of astrocytomas, oligodendrogliomas, and secondary glioblastomas [3]. IDH mutant tumors have distinctive genetic and clinical features, and patients with such mutations have better outcome than those with wild-type IDH [3, 7]. Ki-67 is a reliable indicator of cancer cell proliferation activity and is used for routine clinical investigation, and it predicts worse prognosis for patients with glioma [7–9]. Recent studies have defined the molecular classification of gliomas for clinical use [4, 7], but we still look forward to a more convenient method for predicting prognosis based on IDH1/2 mutation status and Ki-67 expression level.

Herein, we explored IDH1/2 mutation (IDH1/2 mut) and its association with Ki-67 expression in primary tumor samples from a large Chinese Glioma Genome Atlas (CGGA) cohort of 703 gliomas (see Supplementary Table S1). The independent prognostic impact of the two biomarkers on patients with glioma [9, 10] was validated using survival analysis. Then, we applied the two biomarkers to predict diagnosis and prognosis in our glioma classification model.

## RESULTS

### Both IDH1/2 mutation and Ki-67 expression level were important prognostic factors of gliomas

**Figure 1:**
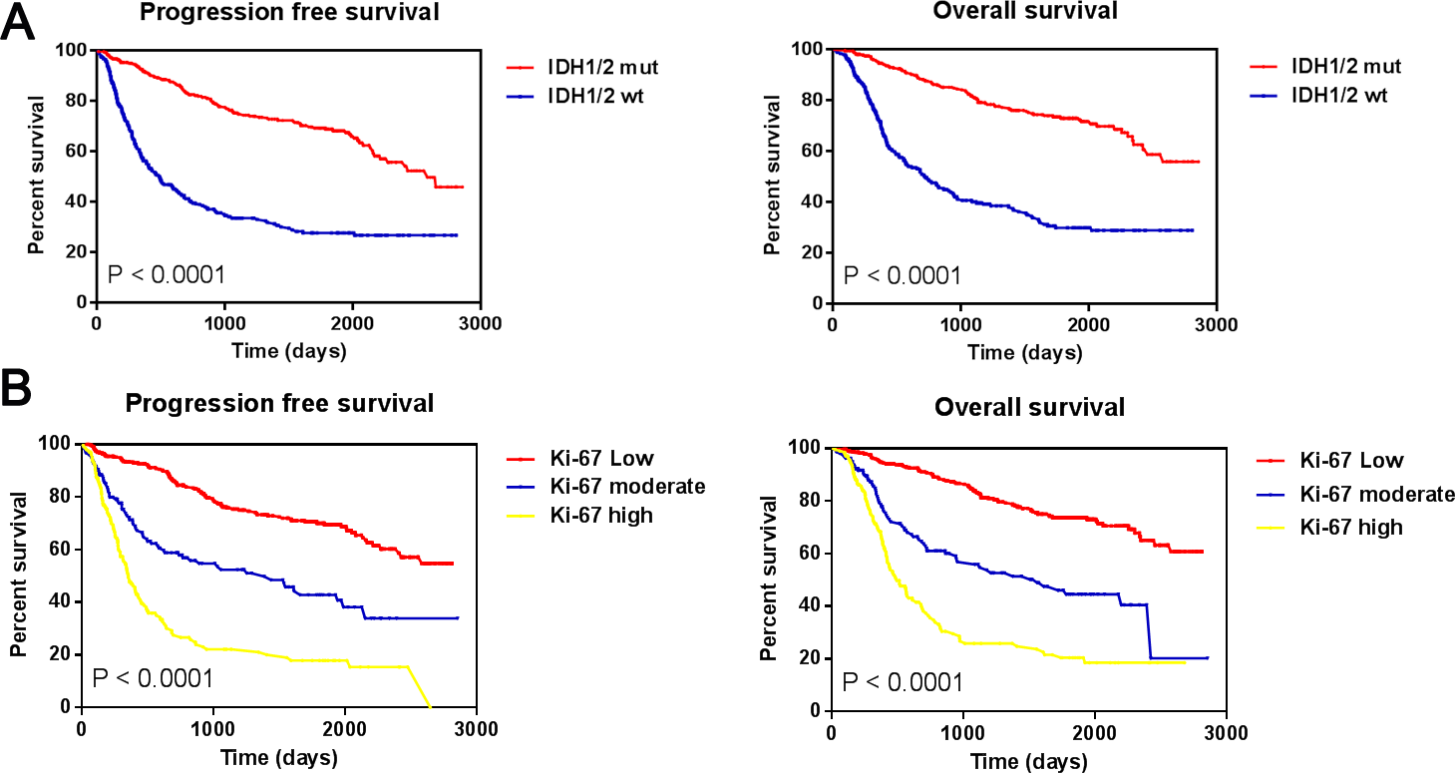
Progression free survival and overall survival among all 703 gliomas **A.** Progression free survival and overall survival among all 703 gliomas stratified by IDH1/2 mutant status; **B.** Progression free survival and overall survival among all 703 gliomas stratified by relative Ki-67 expression level.

Recent investigations implicated IDH1/2 mutation in the classification of biologically distinct groups of gliomas, and indicated improved outcome [11–13]. Therefore, we used Kaplan–Meier curves to evaluate the outcome of 703 patients with glioma following stratification by IDH1/2 mutation status (Figure 1A). IDH mut was associated with longer survival relative to wild-type IDH (IDH1/2 wt) (Median overall survival, OS = 1410 vs. 461 days; log-rank test, *P* < 0.0001). Previously [7, 8], we had classified Ki-67 expression in patients into three levels (low, moderate, high). High Ki-67 expression was strongly associated with shorter progression-free survival (PFS) and OS as compared to moderate Ki-67 expression (Median OS = 381 vs. 619 days). Moderate Ki-67 expression had worse prognosis than low Ki-67 expression (Median OS = 619 vs. 1592 days; log-rank test, *P* < 0.0001). Overall, higher Ki-67 expression was associated with shorter PFS and OS (Figure 1B). Taken together, both IDH1/2 mutation and Ki-67 expression level are reliable prognostic markers of gliomas.

### Decreased Ki-67 expression was prevalent in IDH1/2 mut gliomas

**Figure 2:**
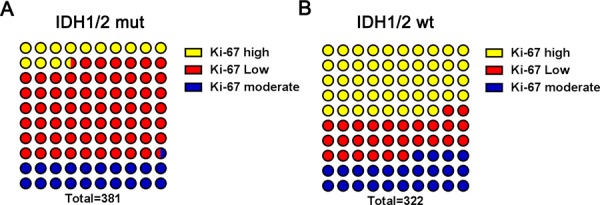
Distribution of low, moderate and high Ki-67 expression among IDH1/2 mut and IDH1/2 wt group **A.** Ki-67 expression was determined in IDH1/2 mut group; **B.** Ki-67 expression was determined in IDH1/2 wt group.

There were 381 samples in the IDH1/2 mut group, which included 250, 79, and 52 low- moderate-, and high–Ki-67 expression gliomas, respectively. The IDH1/2 wt group (*n* = 322 samples), was comprised of 89, 78, and 155 low- moderate-, and high–Ki-67 expression gliomas, respectively. Figure 2 shows that 65.62% (250/381) and 27.64% (89/322) of the IDH1/2 mut and IDH1/2 wt groups, respectively, expressed low Ki-67 (*P* < 0.0001, chi-square test) (Figure 2). The distribution of gliomas with high Ki-67 expression was the inverse of this. The proportion of high Ki-67 expression in the IDH1/2 mut and IDH1/2 wt group was 13.65% (52/381) and 48.14% (155/322), respectively (*P* < 0.0001, chi-square test). No statistical significance was detected in the distribution of moderate Ki-67 between the IDH1/2 mut and IDH1/2 wt groups (*P* = 0.378, chi-square test). These findings confirm a strong correlation between IDH mutation status and Ki-67 expression level, suggesting that low Ki-67 expression is characteristically present in gliomas with IDH mutation.

### Patients with low Ki-67 expression did not survive significantly longer than moderate Ki-67 expression in IDH1/2 mutant group

**Figure 3:**
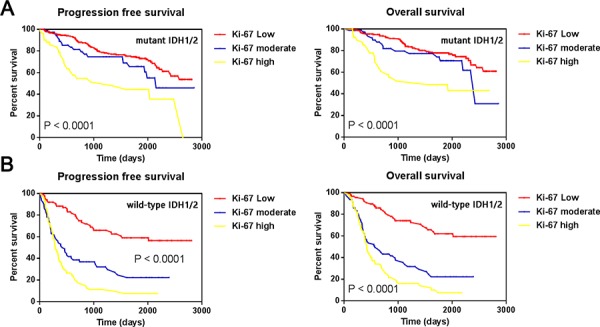
Kaplan-Meier estimates of survival for all 703 gliomas **A.** Progression free survival and overall survival among IDH1/2 mutant tumors stratified by relative Ki-67 expression level; **B.** Progression free survival and overall survival among IDH1/2 wild type tumors stratified by relative Ki-67 expression level.

Olar demonstrated a statistical interaction between IDH mutation and the mitotic index, suggesting that the impact of cellular proliferation on clinical outcome was dependent on IDH mutation status in patients with gliomas [4]. To further understand the effect of Ki-67 expression on prognoses among different IDH mutation statuses, we classified the 703 samples as IDH1/2 mut (Figure 3A) or IDH1/2 wt (Figure 3B). As expected, higher Ki-67 expression in the IDH wt tumors was associated with shorter PFS and OS (Median OS of high, moderate and low Ki-67 = 355, 434, and 1234 days, respectively; log-rank test, *P* < 0.0001). Among the IDH mut tumors, high Ki-67 expression was strongly associated with the shortest PFS and OS (median OS = 566 days; log-rank test, *P* < 0.0001); interestingly, the prognosis of the low and moderate Ki-67 expression groups were not statistically different (median OS = 1699 and 786 days, respectively; log-rank test, *P* = 0.158). Notably, low Ki-67 expression did not necessarily predict better prognosis than moderate Ki-67 expression among the IDH1/2 mut gliomas (Figure 3A).

### IDH1/2 mutation could be used to sub-classify gliomas in combination with Ki-67 expression

**Figure 4:**
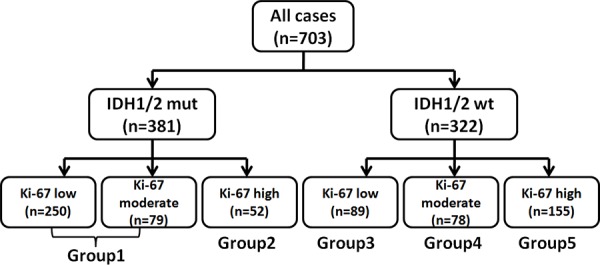
Model for classification of gliomas based on molecular markers IDH1/2 mutant tumors with low and moderate Ki-67 expression was termed as Group 1, IDH1/2 mutant tumors with high Ki-67 expression as Group 2, IDH1/2 wt tumors with low Ki-67 expression as Group 3, IDH wt tumors with moderate Ki-67 expression as Group 4, and IDH wt tumors with high Ki-67 expression was defined as Group 5.

To further detail the glioma molecular classification, we designed a glioma classification model based on IDH1/2 mutation status and Ki-67 expression level (Figure 4). We classified gliomas as IDH1/2 mut or IDH1/2 wt, and then designated IDH1/2 mut with high Ki-67 expression as Group 2, IDH1/2 wt with low Ki-67 expression as Group 3, IDH1/2 wt with moderate Ki-67 expression as Group 4, and IDH1/2 wt with high Ki-67 expression as Group 5. IDH1/2 mut with low and moderate Ki-67 expression were combined into Group 1 due to their similar prognoses, and accounted for 86.35% (329/381) of patients with IDH1/2 mutation and 46.80% (329/703) of all recruited patients.

**Figure 5:**
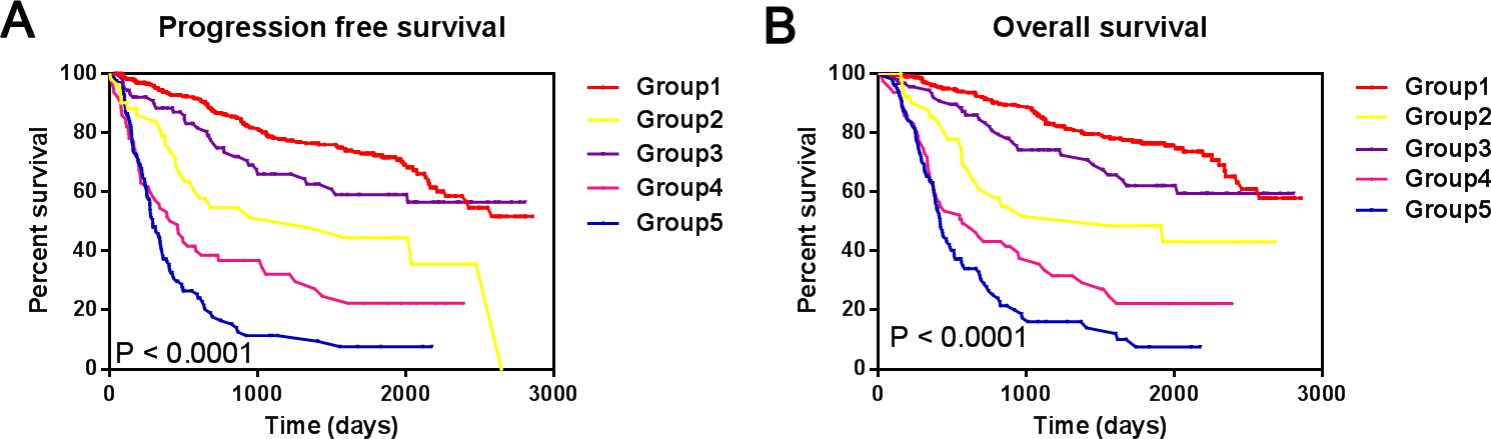
Progression free survival and overall survival among the new five groups **A.** Progression free survival among the new five groups stratified by combined IDH mutation and Ki-67 expression status; **B.** overall survival among the new five groups stratified by combined IDH mutation and Ki-67 expression status.

As shown in Figure 5, Group 1 patients had the longest PFS and OS, whereas Group 5 patients had the shortest (median OS = 1527 vs. 355 days; log-rank test, *P* < 0.0001). These findings confirm the premise that different glioma oncobiology, such as different IDH1/2 mutation status and Ki-67 expression levels, are associated with discriminating prognosis [4]. However, patients with IDH1/2 wt gliomas and low Ki-67 expression survived longer than those with IDH1/2 mut gliomas and high Ki-67 expression (Median OS =1234 vs. 566 days), which contradicts the findings in Figure 1A. Patients with IDH1/2 mut gliomas and high Ki-67 expression survived longer than did those with IDH1/2 wt gliomas and moderate Ki-67 expression (Median OS = 566 vs. 434 days), which contradicts the findings in Figure 1B. Therefore, we conclude that classification combining IDH mutation with Ki-67 expression levels could represent a more precise biological property and prognosis compared to classification using IDH mutation status or Ki-67 expression level separately.

## DISCUSSION

IDH1/2 mutations were first reported in 2009, occurring in about 80% of grade II–III gliomas and secondary glioblastoma multiforme (GBM) [13]. IDH1/2 mutations are early and common events in the etiopathology of astrocytomas, oligodendrogliomas, and oligoastrocytomas, and they are associated with increased DNA methylation, and termed the glioma-CpG island methylator phenotype (G-CIMP) [4]. Wild-type IDH produces α-ketoglutarate, whereas mutant IDH1/2 encodes mutant IDH1/2 proteins. Mutant IDH1/2 proteins have altered substrate specificity and they produce more D-2-hydroxyglutarate, which acts as an oncometabolite [14–16]. Acquisition of IDH1 mutations, followed by compatible molecular changes such as TP53 mutation or 1p/19q codeletion in a common tumor progenitor cell, can lead to gliomagenesis [17]. Although IDH1/2 mutations might not directly trigger tumorigenesis, they increase the risk of other tumor-promoting mutations that cooperate with IDH mutations to induce gliomagenesis [18]. IDH mutation status is stable, while additional molecular events, such as allelic loss of 1p/ 19q (LOH 1p/19q) or TP53- mutations, accumulate during the progression of low-grade glioma to secondary high-grade glioma [19]. Clinically, IDH mut tumors are associated with longer OS as compared to IDH wt tumors among most glioma entities [4, 7]. Hartmann et al. reported that glioblastoma patients with mutant IDH had better outcomes than that of patients with grade III anaplastic astrocytoma with wild-type IDH [11]. All this suggests that IDH mutations correlate with glioma etiopathology and act as a powerful prognostic factor among patients with gliomas. It is not surprising that current findings suggest that glioma subtypes can be separated following stratification by IDH mutation status [11, 14]. In comparison with IDH1/2 mutations being widely considered a key development in the early stage of astrocytic tumors, increasing Ki-67 expression is considered the terminal event in glioma progression [8, 9, 20, 21]. Ki-67 is a nuclear protein and is frequently considered an indicator of cellular proliferation [22]. Similar to IDH mutation, Ki-67 expression is also an independent prognostic factor in glioma [9]. Recent work has reported that high Ki-67 expression is dominant in IDH1/2 wt gliomas [7] and that low Ki-67 expression is associated with IDH1 mutations in primary GBMs [20]. These findings demonstrate the relationship between Ki-67 expression and IDH1/2 mutations in gliomas.

We classified 703 gliomas according to IDH mutation status and Ki-67 expression level regardless of morphological grading. As shown in Supplementary Table S2, we examined groups based on IDH/Ki-67 and WHO grades in multivariate Cox proportional hazard regression analyses, and we found that IDH/Ki-67 could define novel prognostic groups independent of WHO grade. Our molecular classification model detected significantly different prognoses among the five subtypes. Group 1 patients had the best prognosis, whereas Group 5 patients had the worst. That low Ki-67 expression did not differ significantly from moderate Ki-67 expression in Group 1 in terms of clinical outcome could be attributed to the larger effect size of IDH mutation on prognosis among IDH mut tumors. Given the majority of samples with low (*n* = 250) and moderate (*n* = 79) Ki-67 expression among the IDH mut tumors, this effect appeared relatively robust. We also found that patients with IDH1/2 mut gliomas and high Ki-67 expression (Group 2) had worse clinical outcome than did patients with DH1/2 wt gliomas and low Ki-67 expression (Group 3). We speculate that the flourishing cellular proliferation activity in Group 2 contributed to the worse prognosis.

Cai et al. used *ATRX* mRNA expression combined with IDH1/2 mutation status and Ki-67 expression to refine the molecular classification of 169 astrocytic tumors [7]. Olar et al. employed IDH1/2 mutation combined with 1p/19q codeletion and the mitotic index to categorize 558 grade II–III diffuse gliomas [4]. We combined IDH1/2 mutation status with Ki-67 expression level to describe the biological properties and prognosis for each patient quickly and precisely in 703 gliomas.

In summary, our results demonstrate that combining IDH1/2 mutation status with Ki-67 expression levels can be used to define five glioma subgroups regardless of the conventional WHO grade. This approach further characterizes the distinct glioma biological properties and clinical outcome and can act as a complementary description to conventional glioma classification.

## MATERIALS AND METHODS

### Patients and samples

All glioma samples included in our study were from the Chinese Glioma Genome Atlas (CGGA). The patients underwent surgical resection between January 2006 and December 2010. Patients were eligible for the study if the diagnosis of glioma was established histologically according to the 2007 WHO classification. Tumor tissue samples were obtained by surgical resection before treatment with radiation and chemotherapy. This study was approved by the institutional review boards of the hospitals, and written informed consent was obtained from all patients. The following data were collected: WHO grade (as per original pathology report confirmed with H&E central review per current 2007 WHO grading criteria), survival status (alive or dead), IDH1/2 mutational status, Ki-67 expression, overall survival (OS), and progression-free survival (PFS). Data sets are provided in Supplementary Table S1.

### DNA pyro-sequencing for IDH1/2 mutation

Pyro-sequencing was performed as ref [7]. Briefly, genomic DNA was extracted from frozen tissues with a QIAamp DNA Mini Kit (Qiagen) according to the manufacturer's protocol. DNA concentration and quality were measured using a Nano-Drop ND-1000 spectrophotometer (NanoDrop Technologies, Houston, TX). Pyrosequencing of IDH1/2 mutations was supported by Gene-tech (Shanghai, China) and performed on a Pyro-Mark Q96 ID System (Qiagen, Valencia, Calif). The primers 5′-GCT TGT GAG TGG ATG GGT AAA AC-3′, 5′-Biotin-TTG CCA ACA TGA CTT ACT TGA TC- 3′ for IDH1 and 5′-ATC CTG GGG GGG ACT GTC TT-3′, 5′- Biotin-CTC TCC ACC CTG GCC TAC CT-3′ for IDH2 were used for PCR amplification, and the primers 5′-TGG ATG GGT AAA ACC T-3′ for IDH1 and 5′-AGC CCA TCA CCA TTG-3′ for IDH2 were used for sequencing.

### Immunohistochemistry

Immunohistochemistry was performed according to our previous report [7, 8]. Anti-ki-67 at a dilution of 1:100. The staining intensity was jointly scored by two pathologists without knowledge of clinical information on a scale of 0–3, with 0=no or rare occurrence of staining, 1 ≤ 10% of cells positively stained, 2 = 10–30% of cells positively stained, 3 ≥ 30% of cells positively stained. Score 0–1, Score 2 and Score 3 indicated low, moderate and high expression of Ki-67. Controls without primary antibody and positive control tissues were included in all experiments to ensure the quality of staining.

### Statistical analysis

Two clinical end-points were used to measure clinical outcome, progression-free survival (PFS) and overall survival (OS). PFS was defined as the time interval between the date of surgery and the date of first recurrence. OS was defined as the time interval between the date of surgery and the date of death. Kaplan-Meier survival analysis was used to estimate the survival distributions, and the log-rank test was used to assess the statistical significance between stratified survival groups using GraphPad Prism 5.0 statistical software. A two-sided *P* value of < 0.05 was regarded as significant.

## SUPPLEMENTARY MATERIAL TABLES




