# Supplementary material for: IDH1/2 mutation status combined with Ki-67 labeling index defines distinct prognostic groups in glioma
Source: Oncotarget. 2015 Aug 18;6(30):30232–8. doi: 10.18632/oncotarget.4920 (PMC4745793; doi:10.18632/oncotarget.4920)
Supplement: Supplementary file 1 [file oncotarget-06-30232-s001.pdf]

SUPPLEMENTARY TABLES

Supplementary Table S1: Clinical characteristics of cases used in the present study

Supplementary Table S2: Cox proportional hazard regression analyses of molecular subgroups based on IDH/Ki-67 and WHO grades in relation to prognosis in 703 patients with glioma

Variable	Univariable Regression			Multivariable Regression		
	HR	95% CI	*p* Value	HR	95% CI	*p* Value
**Progression-Free Survival**						
WHO grades	3.15	2.74–3.62	<0.001	2.42	2.03–2.88	<0.001
Molecular subgroups	1.70	1.58–1.83	<0.001	1.27	1.16–1.40	<0.001
**Overall Survival**						
WHO grades	3.21	2.77–3.71	<0.001	2.39	1.99–2.88	<0.001
Molecular subgroups	1.75	1.61–1.89	<0.001	1.30	1.18–1.44	<0.001
